# Use of a Novel Grammatical Inference Approach in Classification of Amyloidogenic Hexapeptides

**DOI:** 10.1155/2016/1782732

**Published:** 2016-03-09

**Authors:** Wojciech Wieczorek, Olgierd Unold

**Affiliations:** ^1^Faculty of Computer Science and Materials Science, University of Silesia, Ulica Zytnia 12, 41-200 Sosnowiec, Poland; ^2^Department of Computer Engineering, Faculty of Electronics, Wroclaw University of Science and Technology, Wybrzeże Wyspianskiego 27, 50-370 Wroclaw, Poland

## Abstract

The present paper is a novel contribution to the field of bioinformatics by using grammatical inference in the analysis of data. We developed an algorithm for generating star-free regular expressions which turned out to be good recommendation tools, as they are characterized by a relatively high correlation coefficient between the observed and predicted binary classifications. The experiments have been performed for three datasets of amyloidogenic hexapeptides, and our results are compared with those obtained using the graph approaches, the current state-of-the-art methods in heuristic automata induction, and the support vector machine. The results showed the superior performance of the new grammatical inference algorithm on fixed-length amyloid datasets.

## 1. Introduction

Grammatical inference (GI) is an intensively studied area of research that sits at the intersection of several fields including formal languages, machine learning, language processing, and learnability theory. The main task of the field is about finding some unknown rule when given some elements: examples and counterexamples. This presentation of elements may be finite (in practice) or infinite (in theory). As this study will be especially focused on obtaining a regular expression from finite positive and negative data, the various models of incremental learning and their decidability questions have not been mentioned. The book by de la Higuera [1] can be of major help on such theoretical aspects of grammatical inference.

Here and subsequently *S* = (*S*_+_, *S*_−_) stands for a sample where *S*_+_ is the set of examples and *S*_−_ is the set of counterexamples over a fixed alphabet Σ. Our aim is to obtain a compact description of a finite language *L* satisfying all the following conditions: (i) *L* ⊂ Σ^+^, (ii) *S*_+_⊆*L*, and (iii) *S*_−_∩*L* = *∅*. We will consider a star-free regular expression (i.e., without the Kleene closure operator) as the compact description of a language *L*. It is worthy to emphasize that such a formulation of an induction problem is justified by intended applications in bioinformatics. A sample in biological or medical domains consists of positive and negative objects (mainly proteins) with certain properties, whereas a star-free regular expression may serve to predict new objects. The data explored by Tian et al. [2] and Maurer-Stroh et al. [3] are good illustrations. They consist of examples and counterexamples of amyloids, that is, proteins which have been associated with the pathology of more than 20 serious human diseases. In the experimental part of the present paper, we are going to undertake an examination of binary classification efficiency for selected real biological/medical data. By binary classification, we mean mapping a string to one out of two classes by means of induced regular expressions (regex). For classification, especially for two-class problems, a variety of measures has been proposed. Since our experiments lie in a (bio)medical context, the Matthews Correlation Coefficient is regarded as a primary score, as the goal of this whole process is to predict new strings that are likely to be positive.

There is a number of closely related works to our study. Angluin showed that the problem of inferring minimum-size regular expression satisfying (i), (ii), and (iii) remains NP-complete even if a regex is required to be star-free (containing no “*∗*” operations) [4]. In our previous work [5] similar bioinformatics datasets have been analyzed, but with different acceptors—directed acyclic word graphs. Some of classical automata learning algorithms like ECGI [6], *k*-RI [7], and *k*-TSSI [8] could be applied to the problem, but they do not make use of counterexamples. Many authors advocated the benefit of viewing the biological sequences as sentences derived from a formal grammar or automaton. As a good bibliographical starting point, see articles by Coste and Kerbellec [9], Sakakibara [10], and Searls [11]. In connection with this problem of data classification, it is worth remembering that there is a field of computer science that can be also involved, namely, machine learning (ML), which includes such methods as classification trees, clustering, the support vector machine [12], and rough sets [13]. All above-mentioned ML methods are aimed at compact description of input data, though in various ways. In view of our applications, they have, however, a drawback. The problem is that they are not suited for variable-length data.

In the present algorithm a star-free regular expression (SFRE) is achieved based on a learning sample containing the examples and counterexamples (these examples and counterexamples are also called positive and negative words). It is a two-phase procedure. In the first phase an initial graph is built in order to reveal possible substring interchanges. In the second phase all maximal cliques of the graph are yielded to build a SFRE. We have implemented our induction algorithm of a SFRE and started applying it to a real bioinformatics task, that is, classification of amyloidogenic hexapeptides. Amyloids are proteins capable of forming fibrils instead of the functional structure of a protein [14] and are responsible for a group of diseases called amyloidosis, such as Alzheimer's, Huntington's disease, and type II diabetes [15]. Furthermore, it is believed that short segments of proteins, like hexapeptides consisting of 6-residue fragments, can be responsible for amyloidogenic properties [16]. Since it is not possible to experimentally test all such sequences, several computational tools for predicting amyloid chains have emerged, inter alia, based on physicochemical properties [17] or using machine learning approach [18–21].

To test the performance of our SFRE approach, the following six additional programs have been used in experiments: the implementation of the Trakhtenbrot-Barzdin state merging algorithm, as described in [22]; the implementation of Rodney Price's Abbadingo winning idea of evidence-driven state merging [23]; a program based on the Rlb state merging algorithm [24]; ADIOS (for Automatic Distillation of Structure)—a context-free grammar learning system, which relies on a statistical method for pattern extraction and on structured generalization [25]; our previous approach with directed acyclic word graphs [5]; and, as an instance of ML methods, the support vector machine [26].

A rigorous statistical procedure has been applied to compare all the above methods in terms of a correlation between the observed and predicted binary classification (Matthews Correlation Coefficient, MCC). The proposed approach significantly outperforms both GI-based methods and ML algorithm on fixed-length amyloid datasets.

## 2. Materials and Methods

### 2.1. Datasets

The algorithm for generating star-free regular expressions SFRE has been tested over three recently published Hexpepset datasets, that is, Waltz [3], WALTZ-DB [27], and exPafig [5]. The first two databases consist of only experimentally asserted amyloid sequences. Note that the choice of experimental verified short peptides is very limited since very few data are available. The Waltz dataset has been published in 2010 and is composed of 116 hexapeptides known to induce amyloidosis (*S*_+_) and by 161 hexapeptides that do not induce amyloidosis (*S*_−_). The WALTZ-DB has been prepared by the same science team in the Switch Lab from KU Leuven and published in 2015. This dataset expands the Waltz set to total number of hexapeptides of 1089. According to Beerten et al. (2015), additional 720 hexapeptides were derived from 63 different proteins and combined with 89 peptides taken from the literature [27]. In the WALTZ-DB database, 244 hexapeptides are regarded as positive for amyloid formation (*S*_+_) and 845 hexapeptides as negative for amyloid formation (*S*_−_).

SFRE algorithm was also validated and trained on database (denoted by exPafig), which was computationally obtained with Pafig method [2], and then statistically processed [5]. exPafig consists of 150 amyloid positive hexapeptides (*S*_+_) and 2259 negative hexapeptides (*S*_−_). As seen, the database is strongly imbalanced.

### 2.2. An Algorithm for the Induction of a SFRE

#### 2.2.1. Definitions

**Definition 1 .**

Σ will be a finite nonempty set, the* alphabet*. Σ^+^ will denote the set of all nonempty strings over the alphabet Σ. If *s*, *t* ∈ Σ^+^, the concatenation of *s* and *t*, written *st*, will denote the string formed by making a copy of *s* and following it by a copy of *t*. If *A*, *B*⊆Σ^+^, then

(1) $$\begin{array}{c} AB=\left\{\;s\;|\;s=tu\text{ for some }t\in A,\;\;u\in B\;\right\}. \end{array}$$

To simplify the representations for finite languages, we define the notion of star-free regular expressions over alphabet Σ as follows.

**Definition 2 .**

The set of* star-free regular expressions* (SFREs) over Σ will be the set of strings *R* such that

(1) *∅* ∈ *R* which represents the empty set;
(2) Σ⊆*R*; each element *a* of the alphabet represents language {*a*};
(3) if *r*_*A*_ and *r*_*B*_ are SFREs representing languages *A* and *B*, respectively, then (*r*_*A*_ + *r*_*B*_) ∈ *R* and (*r*_*A*_ *r*_*B*_) ∈ *R* representing *A* ∪ *B*, *AB*, respectively, where the symbols (, ), + are not in Σ.

We will freely omit unnecessary parentheses from SFREs assuming that concatenation has higher priority than union. If *r* ∈ *R* represents language *A*, we will write *L*(*r*) = *A*.

**Definition 3 .**

A* sample S* over Σ will be an ordered pair *S* = (*S*_+_, *S*_−_) where *S*_+_, *S*_−_ are finite subsets of Σ^+^ and *S*_+_∩*S*_−_ = *∅*. *S*_+_ will be called the* positive part of S*, and *S*_−_ the* negative part of S*. A star-free regular expression *r* is* consistent* (or* compatible*) with a sample *S* = (*S*_+_, *S*_−_) if and only if *S*_+_⊆*L*(*r*) and *S*_−_∩*L*(*r*) = *∅*.

**Definition 4 .**

A* graph G* is a finite nonempty set of objects called* vertexes* together with a (possibly empty) set of unordered pairs of distinct vertexes of *G* called* edges*. The vertex set of *G* is denoted by *V*(*G*), while the edge set is denoted by *E*(*G*). The edge *e* = {*u*, *v*} is said to* join* the vertexes *u* and *v*. If *e* = {*u*, *v*} is an edge of a graph *G*, then *u* and *v* are* adjacent vertexes*. In a graph *G*, a* clique* is a subset of the vertex set *C*⊆*V*(*G*) such that every two vertexes in *C* are adjacent. By definition, a clique may be also composed of only one vertex. If a clique does not exist exclusively within the vertex set of a larger clique, then it is called a* maximal clique*.

**Definition 5 .**

Let Σ be an alphabet and let *G* be a graph. Suppose that every vertex in *G* is associated with an ordered pair of nonempty strings over Σ; that is, *V*(*G*) = {*v*_1_, *v*_2_,…, *v*_*n*_}, where *v*_*i*_ = (*u*_*i*_, *w*_*i*_) ∈ Σ^+^ × Σ^+^ for 1 ≤ *i* ≤ *n*. Let *C* = {*v*_*i*_1__, *v*_*i*_2__,…, *v*_*i*_*m*__} be a clique in *G*. Then

(2) $$\begin{array}{c} r\left(\;C\;\right)=\left(\;u_{i_{1}}+u_{i_{2}}+\cdots +u_{i_{m}}\;\right)\left(\;w_{i_{1}}+w_{i_{2}}+\cdots +w_{i_{m}}\;\right) \end{array}$$

is a star-free regular expression over Σ* induced by C*.

For the simplicity's sake, we also denote the set *L*(*u*_*i*_1__ + ⋯+*u*_*i*_*m*__) = {*u*_*i*_1__,…, *u*_*i*_*m*__} by *U* and the set *L*(*w*_*i*_1__ + ⋯+*w*_*i*_*m*__) = {*w*_*i*_1__,…, *w*_*i*_*m*__} by *W* in the context of *C*.

#### 2.2.2. The Algorithm

In this section, we are going to show how to generate a SFRE compatible with a given sample. These expressions do not have many theoretical properties but have marvelous accomplishment in the analysis of some bioinformatics data in terms of classification quality.

Let *S* = (*S*_+_, *S*_−_) be a sample over Σ in which every string is at least of length 2. Construct the graph *G* with vertex set

(3) $$\begin{array}{c} V\left(\;G\;\right)=\overset{}{\underset{s\in S_{+}}{⋃}}\left\{\;\left(\;u,w\;\right)\;|\;s=uw,\;\;u,w\in \Sigma^{+}\;\right\} \end{array}$$

and with edge set *E*(*G*) given by

(4) u,w,x,y∈EG⟺u=x,  uy∉S−,  xw∉S−.

Next, find a set of cliques *𝒞* = {*C*_1_, *C*_2_,…, *C*_*k*_} in *G* such that *S*_+_⊆∑_*i*=1_^*k*^ *r*(*C*_*i*_). For this purpose one can take advantage of an algorithm proposed by Tomita et al. [28] for generating all maximal cliques. Although it takes *O*(*n*3^*n*/3^) time in the worst case for an *n*-vertex graph, computational experiments described in Section 3 demonstrate that it runs very fast in practice (a few seconds for thousands of vertexes). Finally, return the union of SFREs induced by all maximal cliques *𝒞*; that is, *e* = *r*(*C*_1_) + *r*(*C*_2_)+⋯+*r*(*C*_*k*_).

In order to reduce the computational complexity of the induction, instead of Tomita's algorithm, the ensuing randomized procedure could be applied. Consecutive cliques *C*_*i*_ with their catenations *U*_*i*_ *W*_*i*_ are determined until *S*_+_⊆⋃_*i*=1_^*k*^ *U*_*i*_ *W*_*i*_. The catenations emerge in the following manner. In step *i* + 1, a vertex *v*_*s*_1__ = (*u*, *w*) ∈ *V*(*G*) for which *uw* ∉ ⋃_*m*=1_^*i*^ *U*_*m*_ *W*_*m*_ is chosen at random. Let *U*_*i*+1_ = {*u*} and *W*_*i*+1_ = {*w*}. Then sets *U*_*i*+1_ and *W*_*i*+1_ are updated by adding words from the randomly chosen neighbor of *v*_*s*_1__, say *v*_*s*_2__, and subsequently by adding words from the randomly chosen neighbor *v*_*s*_3__ of {*v*_*s*_1__, *v*_*s*_2__}, and so forth. In the end, a maximal clique *C*_*i*+1_ is obtained for which *L*(*r*(*C*_*i*+1_)) = *U*_*i*+1_ *W*_*i*+1_. Naturally, *e* = *r*(*C*_1_) + *r*(*C*_2_)+⋯+*r*(*C*_*k*_) fulfills *S*_+_⊆*L*(*e*), and the whole procedure runs in polynomial time with respect to the input size.

Here are some elementary properties of a resultant expression *e* and the complexity of the induction algorithm.

(i) *S*_−_∩*L*(*e*) = *∅* is implied from (4).
(ii) If all strings in a sample have equal length, let us say *ℓ*, then all strings from *L*(*e*) also are of the same length *ℓ*.
(iii) Let *n* = ∑_*s*∈*S*_ | *s*|. A graph *G*, based on (3) and (4), may be constructed in *O*(*n* ^3^) time. Determining a set of cliques *𝒞* and corresponding regular expressions *r*(*C*_1_), *r*(*C*_2_),…, *r*(*C*_*k*_) also takes no more than *O*(*n* ^3^) time, assuming that the graph is represented by adjacency lists. Thus, the overall computational complexity is *O*(*n* ^3^).

#### 2.2.3. An Illustrative Run

**Figure 1:**
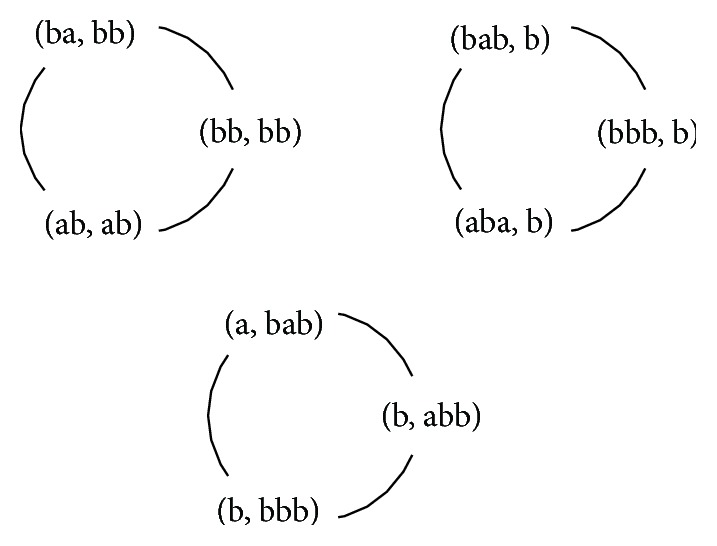
A graph *G* built from a sample *S*, according to definitions (3) and (4).

Suppose S=({bbbb,babb,abab},  {bbba,baba,baaa,abaa,aaba,aaab}) is a sample (one of possible explanations for the input is, each a follows at least one b). A constructed graph *G* is depicted in Figure 1. It has three maximal cliques and regardless of the method—either Tomita's or randomized algorithm was selected—all of them would be determined in this case. The final SFRE induced by the cliques is

(5) e=ab+ba+bbbb+ab+aba+bbb+babb+b+abab+bbb+abb.

Among all words of length four over the alphabet $\left\{a,b\right\}$ it does not accept aaaa,baaa,abaa,bbaa,aaba,baba,abba,bbba,aaab, but accepts baab,abab,bbab,aabb,babb,abbb,bbbb.

### 2.3. Validation with Other Methods

The SFRE classification quality over hexapeptides from three datasets was compared to three state-of-the-art tools for heuristic state merging DFA induction: the Trakhtenbrot-Barzdin state merging algorithm (denoted Traxbar) [22], Rodney Price's Abbadingo winning idea of evidence-driven state merging (Blue-fringe) [23], Rlb state merging algorithm (Rlb) [24], and a context-free grammar learning system ADIOS [25]. The compared set of methods was extended by our previous approach with directed acyclic word graphs (DAWG) [5] and the support vector machine with linear kernel function (SVM) [26].

Trakhtenbrot and Barzdin described an algorithm for constructing the smallest DFA consistent with a complete labeled training set [29]. The input to the algorithm is the prefix-tree acceptor which directly embodies the training set. This tree is collapsed into a smaller graph by merging all pairs of states that represent compatible mappings from string suffixes to labels. This algorithm for completely labeled trees has been generalized by Lang [22] to produce a (not necessarily minimum) machine consistent with a sparsely labeled tree (we used implementations from the archive http://abbadingo.cs.nuim.ie/dfa-algorithms.tar.gz for the Traxbar and for the two remaining state merging algorithms).

The second algorithm that starts with the prefix-tree acceptor for the training set and folds it up into a compact hypothesis by merging pairs of states is Blue-fringe. This program grows a connected set of red nodes that are known to be unique states, surrounded by a fringe of blue nodes that will either be merged with red nodes or be promoted to red status. Merges only occur between red nodes and blue nodes. Blue nodes are known to be the roots of trees, which greatly simplifies the code for correctly doing a merge. The only drawback of this approach is that the pool of possible merges is small, so occasionally the program has to do a low scoring merge.

The idea that lies behind the third algorithm, Rlb, is as follows. It dispenses with the red-blue restriction and is able to do merges in any order. However, to have a practical run time, only merges between nodes that lie within a distance “window” of the root on a breadth-first traversal of the hypothesis graph are considered. This introduction of a new parameter is a drawback to this program, as is the fact that its run time scales very badly with training string length. However, on suitable problems, it works better than the Blue-fringe algorithm. The detailed description of heuristics for evaluating and performing merges can be found in Lang's work [24].

ADIOS starts by loading the corpus (examples) onto a directed graph whose vertexes are all lexicon entries, augmented by two special symbols, begin and end. Each corpus sentence defines a separate path over the graph, starting at begin and ending at end, and is indexed by the order of its appearance in the corpus. Loading is followed by an iterative search for significant patterns, which are added to the lexicon as new units. The algorithm generates candidate patterns by traversing in each iteration a different search path, seeking subpaths that are shared by a significant number of partially aligned paths. The significant patterns are selected according to a context-sensitive probabilistic criterion defined in terms of local flow quantities in the graph. At the end of each iteration, the most significant pattern is added to the lexicon as a new unit, the subpaths it subsumes are merged into a new vertex, and the graph is rewired accordingly. The search for patterns and equivalence classes and their incorporation into the graph are repeated until no new significant patterns are found. The Java implementation of ADIOS made available to us by one of the authors was used in our experiments.

DAWG is a two-phase procedure. In the first phase, an initial directed graph is built in a way that resembles the construction of the minimal DFA, but nondeterminism is also allowed. In the second phase, the directed graph is extended in an iterative process by putting some additional labels onto the existing arcs. The order of putting new labels alters the results; hence a greedy heuristic has been proposed in order to obtain the words most consistent with a sample. We used the same implementation of DAWG as in our earlier work on classification of biological sequences [5].

SVM constructs a hyperplane or set of hyperplanes in a high-dimensional space, which can be used for classification, regression, or other tasks. A good separation is achieved by the hyperplane that has the largest distance to the nearest training data points of any class (so-called functional margin), since, in general, the larger the margin, the lower the generalization error of the classifier. In the experiments, we took advantage of scikit SVM, a machine learning Python library with default parameters [30].

### 2.4. Experiment Design and Statistical Analysis

To estimate the SFRE's and compared approaches' ability to classify unseen hexapeptides repeated stratified *k*-fold cross-validation (cv) strategy was used. Note that holdout method is the simplest kind of cross-validation, but multiple cv is thought to be more reliable than holdout due to its evaluation variance [31]. The simplest form of cross-validation is to split the data randomly into *k* mutually exclusive folds, building a model on all but one fold, and to evaluate the model on the skipped fold. The procedure is repeated *k*-times, each time evaluating the model on the next omitted fold. The overall assessment of the model is based on the mean of *k*-individual evaluations. Since the cv assessment would depend on the random assignment samples, a common practice is to stratify the folds themselves [32]. In a stratified variant of cv, the pseudorandom folds are generated in such a way that each fold contains approximately the same percentage of samples of each class as the whole set. Although the cv is considered as one of the most utilized validation methods, it is well known that cv-based estimators have high variance and nonzero bias [33–36]. It is therefore recommended to use a repeated cross-validation approach [37].

The main problem with (repeated) cv is that the training and test sets are not independent samples. Dietterich [31] found that comparing algorithms on the basis of repeated resampling of the same data can cause very high Type-I errors. It means that statistical hypothesis test, like the standard paired *t*-test, incorrectly rejects a true null hypothesis (so-called false positive). Note that cv can be viewed as a kind of random subsampling. To correct the variance estimate of dependent samples, Nadeau and Bengio [38] proposed the following statistic of the* corrected resampled t-test*:

(6) $$\begin{array}{c} t_{c}=\frac{1/n\sum_{j=1}^{n}x_{j}}{\sqrt{\left(1/n+n_{2}/n_{1}\right){\hat{\sigma }}^{2}}}, \end{array}$$

where *x*_*j*_ is the difference of the performance quality between two compared algorithms on *j*-run (1 ≤ *j* ≤ *n*). We assume that in each run *n*_1_ samples are used for training and *n*_2_ samples for testing. ${\hat{\sigma }}^{2}$ stands for the variance of the *n* differences. This statistic obeys approximately Student's *t*-distribution with *n* − 1 degrees of freedom. The only difference to the standard *t*-test is that the factor 1/*n* in the denominator is by the factor 1/*n* + *n*_2_/*n*_1_. The corrected resampled *t*-test has the Type-I error close to the significance level and—opposite to the McNemar test and the 5 × 2 cv test—low Type-II error (i.e., the failure to reject a false null hypothesis). If we consider test based on *r*-times *k*-fold cv, the statistic

(7) $$\begin{array}{c} t_{c}=\frac{1/\left(k\cdot r\right)\sum_{i=1}^{k}\sum_{j=1}^{r}x_{ij}}{\sqrt{\left(1/\left(k\cdot r\right)+n_{2}/n_{1}\right){\hat{\sigma }}^{2}}} \end{array}$$

has *k* · *r* − 1 degrees of freedom and is called* corrected repeated k-fold cv test*. To detect performance differentiation of compared algorithms we use 10 × 10 cv scheme with 10 (instead of 99) degrees of freedom. This scheme was shown [39] to have excellent replicability. Note that, to perform multiple comparisons involving a control method (i.e., SFRE), we are supposed to control the family-wise error (FWER) [40, 41]. FWER is the probability of making Type-I error when testing many null hypotheses simultaneously. Several methods of relaxing the FWER have been proposed [42]. To keep the probability of rejecting any true null hypothesis small, in our experiments we applied Holm correction [43].

The predictive performance of algorithms was evaluated with the confusion matrix and some of the figures of merit associated with it. First, the following four scores were defined as tp, fp, fn, and tn, representing the numbers of true positives (correctly recognized amyloids), false positives (nonamyloids recognized as amyloids), false negatives (amyloids recognized as nonamyloids), and true negatives (correctly recognized nonamyloids), respectively. The following three figures of merit were considered here, since they are widely used.

The Sensitivity, also known as true positive rate, represents the percentage of correctly identified positive cases and is defined as

(8) $$\begin{array}{c} \text{Sensitivity}=\frac{tp}{\left(tp+fn\right)}. \end{array}$$

Specificity, known is as true negative rate, represents the percentage of correctly identified negative cases and is calculated as

(9) $$\begin{array}{c} \text{Specificity}=\frac{tn}{\left(tn+fp\right)}. \end{array}$$

Matthews Correlation Coefficient is defined as

(10) $$\begin{array}{c} \text{MCC}=\frac{\left(tp\cdot tn-fp\cdot fn\right)}{\sqrt{\left(tp+fn\right)\left(tp+fp\right)\left(tn+fp\right)\left(tn+fn\right)}}. \end{array}$$

Note that several other scores derived from the confusion matrix can be used for estimating the prediction reliability. These three figures of merit, that is, Sensitivity, Specificity, and Matthews Correlation Coefficient, seem to be indispensable for the following reasons. Sensitivity and Specificity tend to be anticorrelated and monitor different aspects of the prediction process. Both of them may range from 0 to +1, where +1 means perfect prediction. Second, Matthews Correlation Coefficient [44] considers both the true positives and true negatives as successful predictions. MCC is always between −1 and +1. A value of −1 indicates total disagreement, 0 random prediction, and +1 perfect prediction. What is important in our case is, MCC is resistant to imbalanced dataset.

## 3. Result and Discussion

**Figure 2:**
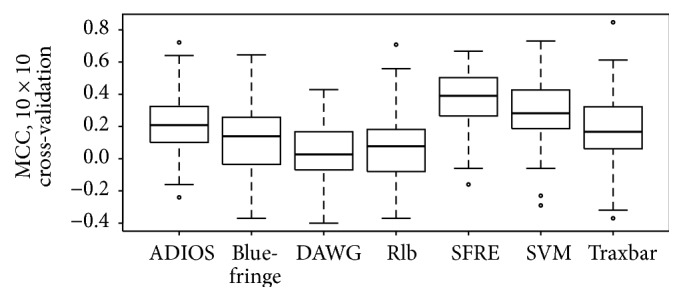
Performance comparison of ADIOS, Blue-fringe, DAWG, Rlb, SFRE, SVM, and Traxbar methods on Waltz database [3]. Boxplots represent the MCC values obtained from 10 × 10 cross-validation. The ratio of *S*_+_/*S*_−_ is 116/161.

**Figure 3:**
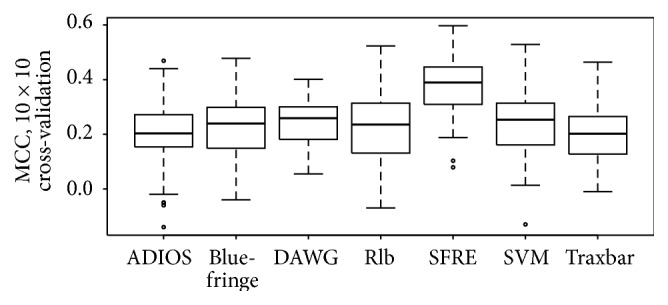
Performance comparison of ADIOS, Blue-fringe, DAWG, Rlb, SFRE, SVM, and Traxbar methods on WALTZ-DB database [27]. Boxplots represent the MCC values obtained from 10 × 10 cross-validation. The ratio of *S*_+_/*S*_−_ is 240/836.

**Figure 4:**
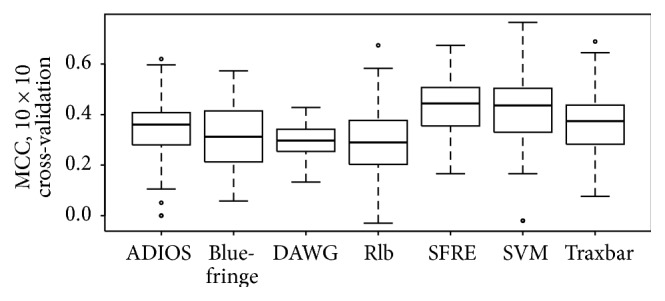
Performance comparison of ADIOS, Blue-fringe, DAWG, Rlb, SFRE, SVM, and Traxbar methods on exPafig database [5]. Boxplots represent the MCC values obtained from 10 × 10 cross-validation. The ratio of *S*_+_/*S*_−_ is 150/2259.

Figure 2 and Table 1, Figure 3 and Table 2, and Figure 4 and Table 3 summarize the performances of the SFRE algorithm and compared methods on Waltz, WALTZ-Db, and exPafig databases, respectively. The figures present boxplots representing the MCC values obtained from 10 × 10 cross-validation, whereas the tables give unadjusted and adjusted by Holm procedure *p* values for the comparison of the SFRE algorithm (the control method) with the remaining algorithms. Note that adjusted *p* for each method and each database is lower than desired level of a confidence *α*, 0.05, in our experiments. These *p* values indicate that there are significant performance differences between SFRE algorithm and compared methods.

SFRE algorithm outperforms all other compared methods in terms of MCC over both experimentally asserted datasets, Waltz and WALTZ-DB, and computationally generated exPafig. It is worth noting that all *p* values except for comparing with SVM algorithm are lower than not only 0.05, but also the often used 0.01, hence confirming the superiority of the SFRE.

Comparative analysis of the three figures of merit (Sensitivity, Specificity, and Matthews Correlation Coefficient) is summarized in Table 4. These quantities are reported for seven compared predictors and three databases (Waltz, WALTZ-DB, and exPafig). Numerical results reported in Table 4 show that SFRE has the highest Average MCC (0.40) followed by SVM (0.31), ADIOS and Traxbar (0.25), Blue-fringe (0.22), and DAWG and Rlb (0.19). Furthermore, SFRE has the highest MCC score compared to the other predictors on each dataset (0.37, 0.38, and 0.44, resp.). Although the results of MCC score seem to be not high (at the level of 0.40), it should be noted that many of the amyloid predictors are reported to have similar or lower values [45]. It is also worth mentioning that all methods have gained the highest MCC values for the computationally generated exPafig dataset.

SFRE has a higher Specificity score than other methods except SVM in case of WALTZ-DB (0.95 to 0.98, resp.) and exPafig databases (both Spe of 1.00). These two predictors have a very good capacity at predicting nonamyloid hexapeptides, with Spe higher than 0.90 for each database. The counterpart is their poor Sensitivity. Concerning Sen score, DAWG, our earlier proposal, has the highest value on each database (0.90, 0.81, and 0.73, resp.). SFRE algorithm showed a low Sensitivity for each tested dataset (0.30, 0.33, and 0.25, resp.).

The evaluation of SFRE on three amyloidogenic hexapeptide datasets revealed its accuracy to predict nonamyloid segments. We showed that the new grammatical inference algorithm gives the best Matthews Correlation Coefficient in comparison to six other methods, including support vector machine.

## 4. Conclusions

In the present paper, the way in which regex induction may support predicting new hexapeptides has been revealed. We, therefore, studied the following problem: given a sample *S* = (*S*_+_, *S*_−_), find a “general” star-free regular expression *e* such that *S*_+_⊆*L*(*e*), *S*_−_∩*L*(*e*) = *∅*, and *L*(*e*) − *S*_+_ contain only strings of “similar characteristics” to those of *S*_+_. To this end, a new GI method has been proposed which is especially suited to the fixed-length datasets. The conducted experiments showed that our algorithm outperforms compared methods in terms of a correlation between the observed and predicted binary classification (MCC) and with real datasets taken from a biomedical domain.

The proposed idea is not free from objections. Among the most serious complications is the exponential computational complexity of generating maximal cliques, which is the second phase of the algorithm. However, it can be overcome by using a proposed randomized procedure instead. Our first experiments on larger datasets uncovered that this is a good direction for the future research.

The high Sensitivity of DAWG approach and high Specificity of SFRE method over tested databases suggest the second direction of future research. These two classifiers could be combined into a metapredictor having, hopefully, both good Sensitivity and Specificity. Such meta-approaches are reported to gain often better results in terms of aggregate indicators (as MCC) than individual predictors [45].

## Figures and Tables

**Table 1:** *p* values for the comparison of the SFRE (the control algorithm) with the other methods on Waltz database. The initial level of confidence *α* = 0.05 is adjusted by Holm procedure.

SFRE versus	Unadjusted *p*	Holm *p*
ADIOS	1.872447*e* − 04	3.744893*e* − 04
Blue-fringe	5.341761*e* − 09	2.136704*e* − 08
DAWG	1.089587*e* − 13	6.537519*e* − 13
RLB	7.529027*e* − 12	3.764514*e* − 11
SVM	9.527442*e* − 03	9.527442*e* − 03
Traxbar	4.257174*e* − 07	1.277152*e* − 06

**Table 2:** *p* values for the comparison of the SFRE (the control algorithm) with the other methods on WALTZ-DB database. The initial level of confidence *α* = 0.05 is adjusted by Holm procedure.

SFRE versus	Unadjusted *p*	Holm *p*
ADIOS	4.904483*e* − 13	1.961793*e* − 12
Blue-fringe	4.495071*e* − 10	4.495071*e* − 10
DAWG	4.838106*e* − 14	2.419053*e* − 13
RLB	3.326237*e* − 11	6.652474*e* − 11
SVM	1.703864*e* − 11	5.111592*e* − 11
Traxbar	9.161256*e* − 16	5.496754*e* − 15

**Table 3:** *p* values for the comparison of the SFRE (the control algorithm) with the other methods on exPafig database. The initial level of confidence *α* = 0.05 is adjusted by Holm procedure.

SFRE versus	unadjusted *p*	Holm *p*
ADIOS	1.501499*e* − 05	4.504496*e* − 05
Blue-fringe	1.019785*e* − 08	4.079139*e* − 08
DAWG	4.319299*e* − 12	2.591579*e* − 11
RLB	7.401268*e* − 11	3.700634*e* − 10
SVM	3.295963*e* − 02	3.295963*e* − 02
Traxbar	1.368667*e* − 04	2.737334*e* − 04

**Table 4:** Performance of compared methods on Waltz, WALTZ-DB, and exPafig databases in terms of Sensitivity (Sen), Specificity (Spe), and Matthews Correlation Coefficient (MCC). The results are ordered by decreasing Average MCC (Ave MCC).

Method	Waltz			WALTZ-DB			exPafig			Ave MCC
	Sen	Spe	MCC	Sen	Spe	MCC	Sen	Spe	MCC	
SFRE	0.30	0.97	0.37	0.33	0.95	0.38	0.25	1.00	0.44	0.40
SVM	0.35	0.90	0.30	0.15	0.98	0.24	0.22	1.00	0.40	0.31
ADIOS	0.36	0.82	0.22	0.64	0.59	0.20	0.51	0.90	0.34	0.25
Traxbar	0.56	0.61	0.17	0.46	0.76	0.20	0.42	0.96	0.37	0.25
Blue-fringe	0.58	0.53	0.11	0.36	0.85	0.23	0.33	0.96	0.32	0.22
DAWG	0.90	0.13	0.04	0.81	0.47	0.24	0.73	0.80	0.30	0.19
Rlb	0.36	0.70	0.07	0.26	0.90	0.22	0.25	0.97	0.29	0.19
